# Effects of proton pump inhibitor use on the esophageal microbial community

**DOI:** 10.1186/s12876-020-01460-3

**Published:** 2020-09-23

**Authors:** Sadia Tasnim, Aaron L. Miller, Daniel C. Jupiter, Catherine F. Hamilton, Gabriel L. Reep, Timothy S. Krill, Richard B. Pyles, Ikenna C. Okereke

**Affiliations:** 1 Division of Cardiothoracic Surgery, University of Texas Medical Branch, 301 University Blvd, Galveston, TX 77555 USA; 2 Department of Microbiology and Immunology, University of Texas Medical Branch, Galveston, TX USA; 3 Department of Preventive Medicine and Population Health, University of Texas Medical Branch, Galveston, TX USA; 4 Division of Gastroenterology, University of Texas Medical Branch, Galveston, TX USA

**Keywords:** Microbiome, Proton pump inhibitor, Dose effect

## Abstract

**Background:**

Changes in the esophageal microbiome correlate with esophageal disease, but the effects of proton pump inhibitor (PPI) drugs are incompletely characterized. Our objective was to identify the effects of PPI use on the microbial community of the esophagus.

**Methods:**

Mucosal biopsies of the distal esophagus were analyzed using a customized esophageal microbiome qPCR panel array (EMB). Patient demographics, use of PPIs, duration of use and dose were recorded.

**Results:**

Fifty-eight patients were included. Mean age was 60.5 years. Ninety percent (52/58) of patients were on PPIs. Mean dose was 42.7 mg. Mean duration of use was 2.5 years. The use of PPIs led to a significant difference in absolute levels of only one organism, *Actinomyces*, in the entire array (*p* < 0.01). Among patients who used proton pump inhibitors, there was no significant association between dose and absolute levels of any organism. Similarly, there was no association between duration of use and absolute levels of any organism.

**Conclusions:**

PPI use does not seem to cause significant changes in the distal esophageal microbial community. Future studies with larger sample sizes and esophageal pH testing should be performed to determine the level of acidity and its relationship to the microbial community.

## Background

Proton pump inhibitors (PPIs) are one of the most commonly used medications worldwide. They block the hydrogen-potassium ATPase receptor and inhibit gastric acid secretion by the parietal cell of the stomach. They are used to treat gastroesophageal reflux disease (GERD), prevent gastric and duodenal ulcers, treat *Helicobacter pylori* infections and many other diseases. PPIs have been known to change the gut microbiome and increase the incidence of *Clostridium difficile*, *Campylobacter* and *Salmonella* infections [1, 2]. However, the interaction between PPIs and the esophageal microbiome has not been properly explored. It has been hypothesized that PPI use can alter the esophageal microbiome in two ways. One potential mechanism is by altering the pH of the distal esophagus secondary to gastric acid production [3]. A second possible mechanism is by directly targeting the P-type ATPase bacterial proton pumps and changing the pH of the bacterial microenvironment [4].

The normal distal esophageal microbiome is dominated by gram-positive organisms, particularly *Streptococcus* [5]*.* A prior study noted a significant increase of organisms from the *Lachnospiraceae, Comamonadaceae*, and *Clostridiaceae* families in the distal esophagus after PPI use [6]. Another study reported a decrease of organisms from the *Comamonadaceae* family and an increase of organisms from the *Clostridiaceae*, *Lachnospiraceae, Microccocaceae, Actinomycetaceae* families after 8 weeks of moderate dose PPI use [4]. In that study there were two additional unidentified families from the *Lactobacillales* and *Gemellales* orders which had different levels after the same PPI use. However, the location in the esophagus where these changes occurred was not noted.

Given the lack of clarity about whether PPI use causes an alteration of the esophageal microflora, and what those changes are, this study was undertaken. Our goal was to evaluate the effects of PPI use, PPI dose, and duration of use on the microorganism community in the distal esophagus.

## Methods

### Study participants

After approval was obtained from the University of Texas Institutional Review Board (IRB# 17–0215), 58 patients were included in the study. All participants were 1) patients undergoing surveillance endoscopy for a known history of Barrett’s esophagus (BE) or 2) patients for whom screening endoscopy was recommended or could be considered based on guidelines from the American College of Gastroenterology. Indications for screening included men or women with chronic symptoms (greater than 5 years) of gastroesophageal reflux disease (GERD) and two or more risk factors for Barrett’s esophagus or esophageal adenocarcinoma. Risk factors included Caucasian race, age ≥ 50 years, chronic GERD symptoms, current or prior history of smoking, central obesity as defined as a waist circumference greater than 88 cm, waist to hip ratio greater than 0.8 or a family history of Barrett’s esophagus or esophageal adenocarcinoma [7]. Patients were enrolled prospectively and consent to participate was obtained voluntarily for each patient. Patients who did not have Barrett’s esophagus or met criteria for screening were not included in this study. Any patient who had been on antibiotic treatment within 3 months of endoscopy were not included in this study.

### Clinical characteristics

Demographic information such as age, gender, ethnicity, body mass index (BMI), smoking status, pack-years, weight change in six to 12 months prior to surveillance endoscopy, presence of Barrett’s esophagus, prior PPI use, dose and number of years of PPI use were recorded. To calculate the dose, the functional equivalents of each type of medication (omeprazole, pantoprazole, lansoprazole) were used for analysis. All but one patient was either on omeprazole or pantoprazole.

### Endoscopy

Prior to its use, the endoscope was sterilized and placed in a sterile container. The endoscope was then inserted into the esophagus and advanced to the gastroesophageal junction without entering the stomach. During the endoscopy, a biopsy of the distal esophagus was used specifically for this study. This biopsy was performed in the distal esophagus and within 2 cm of the gastroesophageal junction. After the biopsy was collected, the endoscope was re-inserted, and the full examination was completed.

### Microflora array

**Fig. 1:**
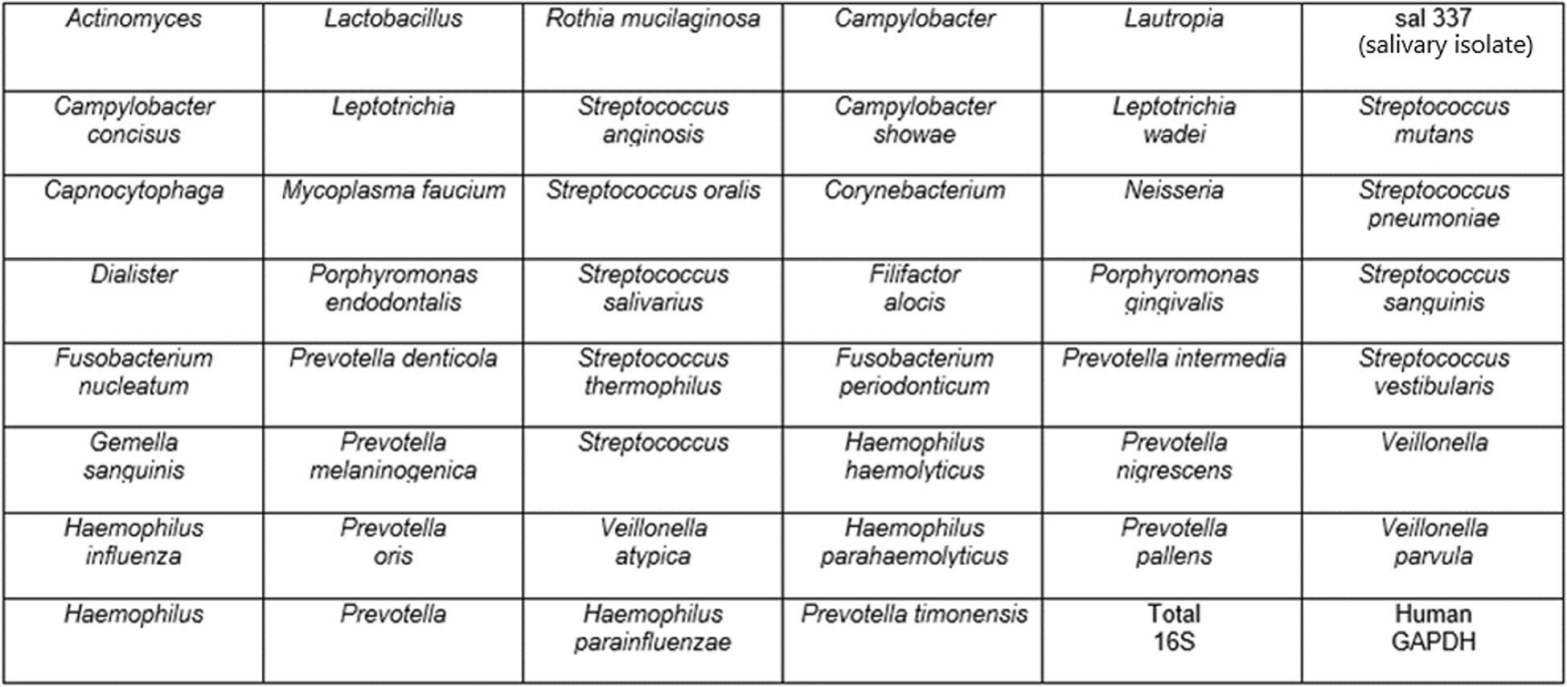
Organisms on EMB array

All biopsies were evaluated using a quantitative polymerase chain reaction (qPCR) panel labeled the Esophageal Microbiome Array (EMB). To construct this array and determine suitable organisms to analyze, a pilot study was performed in which 10 patients with Barrett’s esophagus and 4 patients without Barrett’s esophagus were investigated. In each group, their specimens were pooled and analyzed. The EMB qPCR array (Fig. 1) was developed based on Next-Generation Sequencing data and literature searches to allow higher throughput analyses that produced absolute abundance and more sensitive detection data. Forty-six targeted organisms were selected for the EMB array analysis based on 1) the most prevalent organisms in the pilot study or 2) identified in the literature as possibly being implicated in the development of esophageal disease [8–15].

### DNA extraction

Once obtained during endoscopy, tissue biopsies were placed into sterile Powerbead tubes pre-loaded with 0.1 mm glass beads (Qiagen, Germantown, MD) plus external lysis buffer IVD (200 μL, Roche Applied Science, Indianapolis, IN). Tissues were homogenized at 30 Hz for 5 min using a Tissuelyser II homogenizer (Qiagen). Sample lysates were deposited into individual wells of 96 deep-well processing plates. DNA was subsequently extracted in high-throughput fashion using a Magna Pure 96 instrument employing a Magna Pure 96 DNA and viral small volume-IVD extraction kit according to the manufacturer’s protocol (Roche). After extraction, a portion of the DNA was evaluated using the EMB. The remaining material was archived at -20C.

### Sequencing

Sample sequencing was carried out using a fusion-PCR method. Briefly, fusion-primers were designed in accordance with the manufacturer’s guidelines (Ion Amplification Library Preparation – Fusion Method, Life Technologies, Carlsbad, CA) using Ion Xpress Barcodes linked to 16S gene primer pairs targeting hyper-variable regions 1–8 [16]. Each 25 μl PCR was carried out using: 12.5 μl iQ supermix™ (Bio-Rad, Hercules, CA), 1 μl of both forward and reverse (5 μM) primers, 9.5 μl nuclease-free water and 1 μl of DNA template. DNA from each patient from each sample (uvula swab, proximal esophageal mucosa, distal esophageal mucosa) was used as a template for the creation of subsequent fusion 16S libraries. PCR was completed in a c1000 thermocycler (Bio-Rad) using the following parameters: Cycle 1), 95 C, 3 min, Cycle 2), Step 1—95 C, 45 s; Step 2—Primer-specific annealing temps., 45 s; Step 3—72 C 2:00, repeat 39x; Step 4—72 C for 7:00. PCR products were purified using Qiagen Qiaquick spin-columns and quantified using a spectrophotometer (Bio-Rad). PCR products were then diluted, mixed in equal proportion and sequenced on an Ion Torrent GeneStudio S5 System using Ion 520 sequencing kits together with 520 size chips following the manufacturer’s instructions (Life Technologies).

### Bioinformatics for ion torrent

After generation, sequencing reads were filtered for quality and binned according to Ion Xpress barcode using Ion Torrent Suite software version 5.10.0. Sequencing reads in FASTQ format were further processed using web-based Galaxy software [17]. First, raw FASTQ files were normalized using the FASTQ groomer tool function. Next, each barcoded read was trimmed to remove the primer sequence and subsequently filtered to the expected size of the 16S gene target. After this level of processing, the sequence reads were concurrently compared to the SILVA 16S database using bowtie 2 software [18, 19]. The number of times each sequence matched the database (hit-rate) was recorded. When multiple hits to the same genera or species were made, the number of hits were added accordingly. These numbers were then converted to the percentage of the total to give an overall ratio of the sequenced sample.

### qPCR evaluation by esophageal microbiome Array

qPCR arrays evaluating 46 targets identified in preliminary screens and 2 controls (16S and hGAPDH) were constructed in a 96-well plate format (ThermoFisher Scientific Inc.). Arrays were constructed in 6 × 8 format allowing for evaluation of 2 samples per plate. Each 25 μl PCR was carried out using: 12.5 μl iQ SYBR green supermix™ (Bio-Rad), 1 μl of each forward and reverse (5–10 μM) primer, 9.5 μl nuclease-free water and 1 μl of DNA template. qPCR was completed in a c1000 thermocycler equipped with a CFX™ reaction module (Bio-Rad) using the following parameters: Cycle 1), 95 C, 3 min, Cycle 2), Step 1. 95 C, 30 s, Step 2 annealing 60 C, 30 s, extension 72 C, 30 s repeat 39x, Step 3. 72 C for 2:00, Step 4. Melt-curve 75 C – 89 C, 0.2 C temperature increments with 5-s plate read-time. Fluorescent signal data was collected at the end of each annealing/extension step. Starting quantity values were extrapolated from standard curves of plasmids harboring the PCR targets. Any organism which was below the threshold of detection was categorized as not detected. Mathematical analyses were performed using Excel™ (Microsoft Corp., Redmond, WA).

### Statistical analysis

Absolute levels of organisms were normalized to the level of 16S for each experiment. Organisms which were detected in less than 10 patients were not compared, given the low predictive ability using this model with such few results. Normalized levels of each organism were compared in patients with and without PPI history using independent samples t-tests. To analyze the association of dose and duration of PPI use with normalized levels of each organism, analysis of variance (ANOVA) testing, while adjusting for age, gender, presence of Barrett’s esophagus and smoking history as covariates in each analysis.

Our study inherently recruited patients with Barrett’s esophagus and/or with significant GERD symptoms, as it was difficult to justify endoscopy and mucosal biopsy in patients without symptoms or other reason for endoscopy. To overcome this potential bias, we performed an in-depth analysis on the associations of dose with levels of organisms and duration with levels of organisms. To perform these analyses, only the group with PPI use was examined.

## Results

### Demographics

Demographic data is shown in Table 1. There were 26 patients in the study with Barrett’s esophagus. The remaining 32 patients had GERD, but had no Barrett’s esophagus or adenocarcinoma present. Ninety percent of patients were on PPI medications. There were no statistically significant differences in any demographic variables when comparing patients on PPI medication versus those not taking PPI medication. Among patients who were on PPI medication, average dose was 42.7 mg.

**Table 1:** Demographics

	Patients with PPI use	Patients without PPI use	*P*-value
N	52	6	
Barrett’s esophagus	48% (25/52)	17% (1/6)	0.21
Male	56% (29/52)	17% (1/6)	0.07
Mean age (years)	60.4 (36—83)	60.5 (52—68)	0.99
Mean BMI	30.2 (17.9—40.3)	30.8 (21.0—38.6)	0.82
Mean weight change (kilograms)	Loss of 0.7	Loss of 2.8	0.44
Ethnicity			
Caucasian	79% (41/52)	83% (5/6)	0.80
Hispanic	13% (7/52)	17% (1/6)	
African American	6% (3/52)		
Asian	2% (1/52)		
Presence of hiatal hernia	37% (19/52)	33% (2/6)	0.88
Smoking status			
Current	21% (11/52)	33% (2/6)	0.78
Past	35% (18/52)	33% (2/6)	
Never	44% (23/52)	33% (2/6)	
Mean PPI dose (milligrams)	42.7 (20—80)		
Mean PPI duration (years)	2.5 (1—13)		

### Microbiome detection patterns in PPI users and non-users

Table 2 shows the normalized absolute levels of organism in patients with and without a history of PPI use. There were no statistically significant differences in any demographic variables between the groups. Fifteen of the 46 organisms had more than 10 detections and were included in the analysis. A statistically significant difference was seen only for *Actinomyces* (*p* < 0.01) among the organisms in the array. *Actinomyces* levels were statistically higher in patients who were on PPI medication compared to those not on PPI medication.

**Table 2:** Organism Levels Vs. PPI Use

Organism	PPI use = Y Normalized absolute level of organism	PPI use = N Normalized absolute level of organism	***P***-value
**Actinomyces**	**3.622E-03**	**8.420E-04**	**< 0.01**
Corynebacterium	4.409E-04	1.378E-05	0.06
Dialister	6.930E-03	4.754E-03	0.29
Gemella sanguinis	1.436E-04	1.504E-04	0.96
Haemophilus haemolyticus	8.739E-04	1.951E-03	0.38
Haemophilus parainfluenzae	6.530E-04	1.523E-03	0.08
Leptotrichia	7.233E-04	3.15E-04	0.27
Neisseria	3.399E-03	1.578E-02	0.16
Prevotella	6.650E-02	6.668E-02	0.99
Prevotella melaninogenica	1.393E-04	3.185E-04	0.53
Prevotella pallens	4.071E-04	2.529E-04	0.58
Rothia mucilaginosa	7.118E-04	7.719E-04	0.79
Streptococcus	2.274E-03	2.315E-03	0.44
Streptococcus salivarius	8.072E-04	5.580E-04	0.59
Veillonella	9.729E-04	6.296E-04	0.24

### Microbiome detection pattern versus increasing PPI dose

**Fig. 2:**
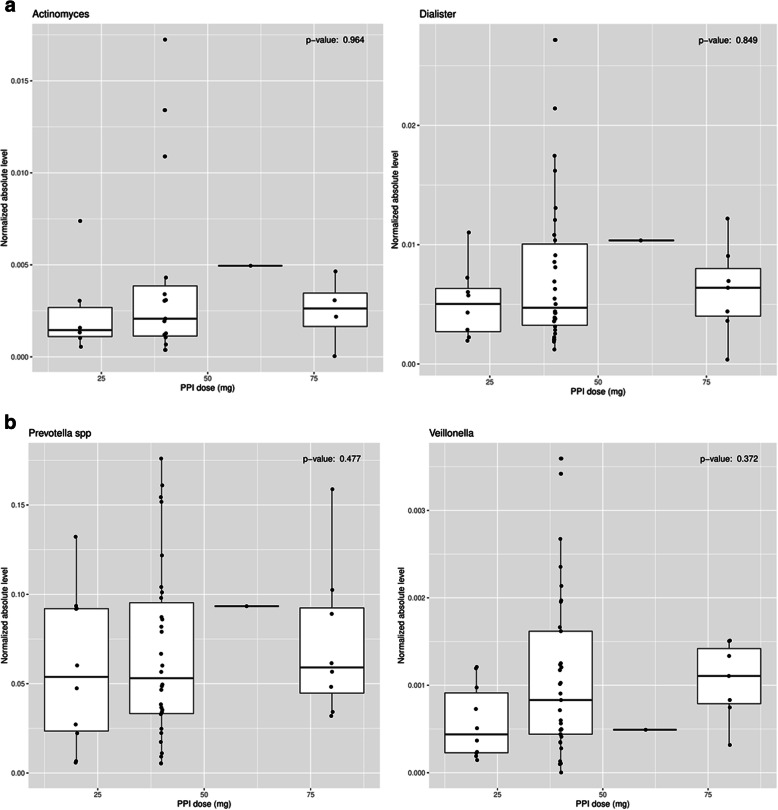
Relationship of PPI dose vs. normalized absolute level of organism for. **a**. *Actinomyces*/*Dialister.* **b***. Prevotella/Veillonella*

When examining the cohort of patients on PPI medication, no organism in the array had its absolute levels affected by the dose of PPI medication. Table 3 shows the lack of significance for level of organism vs. PPI dose for every organism in the array. Fig. 2a-b show the associations of PPI dose to absolute levels for *Actinomyces*, *Dialister*, *Prevotella* and *Veillonella*. These organisms were four of the most prevalent organisms seen in our study, both in patients on PPI medication and patients not on PPI medication.

**Table 3:** Organism level Vs. PPI Dose. No organism had a significant relationship

Organism	***P***-value
Actinomyces	0.96
Corynebacterium	0.17
Dialister	0.85
Gemella sanguinis	0.37
Haemophilus	0.18
Haemophilus haemolyticus	0.79
Haemophilus parainfluenzae	0.75
Leptotrichia	0.97
Neisseria	0.65
Prevotella	0.48
Prevotella melaninogenica	0.15
Prevotella pallens	0.98
Rothia mucilaginosa	0.62
Streptococcus	0.55
Streptococcus salivarius	0.76
Streptococcus vestibularis	0.51
Veillonella	0.37

### Microbiome detection pattern versus increasing PPI duration

**Fig. 3:**
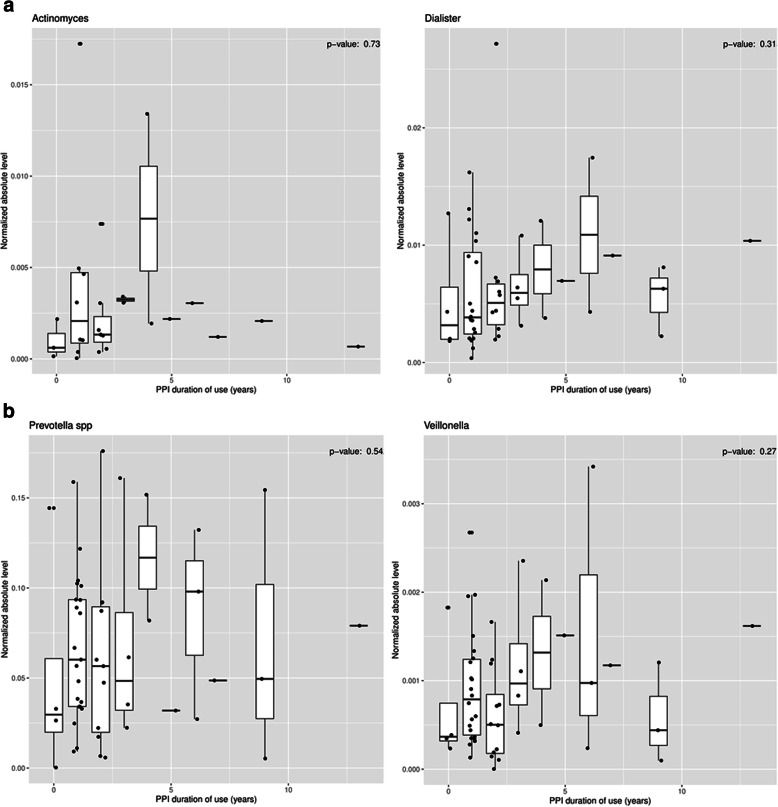
Relationship of PPI duration of use vs. normalized absolute level of organism for. **a**. *Actinomyces*/*Dialister.* **b***. Prevotella/Veillonella*

Similarly, there was no association between years of PPI use and absolute levels of any organisms. Table 4 shows the lack of significance for level of organism vs. PPI duration of use for every organism in the array. Fig. 3a-b show the associations of PPI dose to absolute levels for *Actinomyces*, *Dialister*, *Prevotella* and *Veillonella*.

**Table 4:** Organism level Vs. PPI Duration. No organism had a significant relationship

Organism	***P***-value
Actinomyces	0.50
Corynebacterium	0.43
Dialister	0.38
Gemella sanguinis	0.78
Haemophilus	0.98
Haemophilus haemolyticus	0.52
Haemophilus parainfluenzae	0.74
Leptotrichia	0.86
Neisseria	0.58
Prevotella	0.66
Prevotella melaninogenica	0.85
Prevotella pallens	0.59
Rothia mucilaginosa	0.49
Streptococcus	0.93
Streptococcus salivarius	0.83
Streptococcus vestibularis	0.42
Veillonella	0.34

## Discussion

PPI medications are widely used worldwide for the treatment of GERD. Previous literature has not equivocally shown the effect of PPI use on the microbial community in the gastrointestinal tract [20, 21]. In particular, there have been only very few studies on the effects of PPI use on the esophageal microflora [22]. Considering its wide use and easy availability, the impact of PPI use on the esophageal microflora should be properly analyzed.

Our study showed two important findings. Firstly, of the 46 organisms in the array, only *Actinomyces* showed a difference in levels in patients with and without PPI use. A prior study showed an increase in levels of organisms in the *Actinomycetaceae* family after 8 weeks of PPI use [23]. There is minimal literature or previous investigations about the association of *Actinomyces* and disease in the gastrointestinal tract. *Actinomyces* is associated with dental abscesses and pulmonary actinomycosis [24]. *Actinomyces* is a relatively prevalent organism typically found in the skin, mouth, gastrointestinal tract and genitourinary system.

No other organism beside *Actinomyces* was affected by PPI use. It appears that the microflora changes in the esophagus may be less significant than previously considered. This relative stability has important implications when considering future microbiome studies and the impact of PPI use when designing the methodology. Though further studies are needed, our findings are compelling and demonstrate that PPI use does not modify the distal esophageal microbial community significantly.

Secondly, our study showed that the dose or duration of PPI use does not appear to affect the microbial community in the distal esophagus. We designed our study to examine not only the effects of the PPI use compared to no use, but also to assess whether the amount of exposure affected the levels of organisms found in the esophagus. Previous studies have looked at PPI users versus non-users [25], but our study is novel in that we have shown that amount of exposure also does not appear to affect the levels of organisms in the distal esophagus. We felt that our cohort would be useful to examine this question, as our patients had a wide range of PPI doses from 20 mg to 80 mg. A higher dose should theoretically raise the pH within the lumen of the distal esophagus, but the influence of pH on the microbial community is indeterminate. Furthermore, we did not perform pH testing at the time of mucosal biopsy to determine whether the PPI medication was in fact suppressing acid exposure in the distal esophagus in our patients. Despite these limitations, there was no association between PPI dose and changes in microbial levels in the distal esophagus.

There was also a wide range of duration of PPI use in our cohort. Many studies have described numerous adverse effects of long term PPI use, such as nutrient deficiencies and renal failure [26, 27]. Our patients had a range of use from 1 year to 13 years. Similar to the relationship between dose and microflora in the esophagus, the duration of PPI use did not affect the levels of organisms in the distal esophagus in our cohort.

The lack of alteration of the microbial community with PPI use is an important finding. Previous studies have shown that patients who develop Barrett’s esophagus have a different microbial community in their esophagus than patients without Barrett’s esophagus [28]. It is likely that there are factors other than pH and PPI use which cause this conversion of the esophageal mucosa and increased risk for esophageal cancer. As future studies examine the mechanism by which the mucosal changes occur, other etiologies than pH and PPI use must be considered when creating the experimental designs.

In our study the effect of PPI use on the esophageal microbiome was not as robust as previously predicted. But a limitation of our study is lack of pH testing to determine the effectiveness of the PPI use in each patient. Corresponding pH testing would give more data about the environment of the distal esophagus, but the invasiveness and increased burden of pH testing for most patients would exceed the clinical usefulness. We also could not tell the level of compliance of each patient in taking their PPI medication as prescribed, but this is an inherent limitation of any study which attempts to evaluate the association of PPI use with a particular outcome.

Another limitation of our study is that antibiotics use in our patient population was not considered while analyzing the data for this study. Recent antibiotic use can have a confounding effect on the esophageal microbiome. Given that the overwhelming majority of endoscopies in our patients were performed on an outpatient basis, however, we expect that the rate of antibiotic use was very low in our cohort.

The ideal experiment to determine the effect of PPI use on the microflora community in the distal esophagus would be to perform endoscopy with biopsy before a patient begins a PPI medication. Additional biopsies would then be performed at various time intervals after the introduction of the medication. Though this design would give a clearer understanding of the effects of PPI drugs on organisms in the distal esophagus, clearly there would be no justification for the repetitive endoscopy procedures on the same patient. But we will perform repeat biopsies and analyses on those patients in this study who were not on PPI medication but had treatment begun after the endoscopy.

An ideal experiment also would have equal numbers of patients in the groups who did and did not use PPI medication. But since the majority of patients eligible for surveillance have GERD symptoms, most patients will be on treatment. It would be difficult to justify endoscopy and mucosal biopsy on a healthy control patient solely for research purposes. This is a critical limitation of our study, but it is an inherent design flaw unfortunately. We do feel that we mitigated that problem of uneven groups, however, by performing detailed analyses showing a lack of association between levels of organism versus dose or duration of PPI use. And though there were only few patients in the non-PPI group, there was a wide range of PPI dose in the group of patients who did use PPI medication. The fact that there was no association between dose and levels of organism further supports the relatively limited effect of PPI use on the esophageal microflora.

## Conclusions

PPI use does not significantly change the lower esophageal microbial composition. Increases in dose or duration of PPI use also does not alter the lower esophageal microbiome. Further studies with larger sample sizes and corresponding pH testing can further clarify the effects of PPI on the esophageal microbiome and determine the safety of widespread PPI use.

## Data Availability

The datasets used and/or analyzed during the current study are available from the corresponding author on reasonable request.

## Abbreviations

PPI: Proton pump inhibitor
EMB: Esophageal Microbiome Array
qPCR: Quantitative Polymerase Chain Reaction
GERD: Gastroesophageal Reflux Disease
BMI: Body Mass Index
ANOVA: Analysis of Variance
